# Assessment of left atrial systolic dyssynchrony in paroxysmal atrial fibrillation and heart failure using cardiac magnetic resonance imaging: MESA study

**DOI:** 10.1186/1532-429X-17-S1-P322

**Published:** 2015-02-03

**Authors:** Luisa A Ciuffo, Ravi Sharma, Mohammadali Habibi, Bharath Ambale Venkatesh, Boaz D Rosen, Masamichi Imai, Steven Shea, Robyn McClelland, Colin O Wu, Susan R Heckbert, David Bluemke, Joao A Lima

**Affiliations:** 1 Johns Hopkins, Baltimore, MD, USA; 2 Universidade federal da Bahia, Salvador, Brazil; 3 National Institutes of Health, Bethesda, MD, USA; 4 University of Washington, Washington, DC, USA; 5 Columbia University, New York, NY, USA; 6 Union memorial Hospital, Baltimore, MD, USA

## Background

Left atrial (LA) remodeling in response to cardiovascular and hemodynamic stress may precede atrial fibrillation (AF) and heart failure (HF). We hypothesized that LA systolic synchronous contraction as a functional measure of LA remodeling is deranged in patients with paroxysmal AF and HF.

## Methods

**Figure 1:**
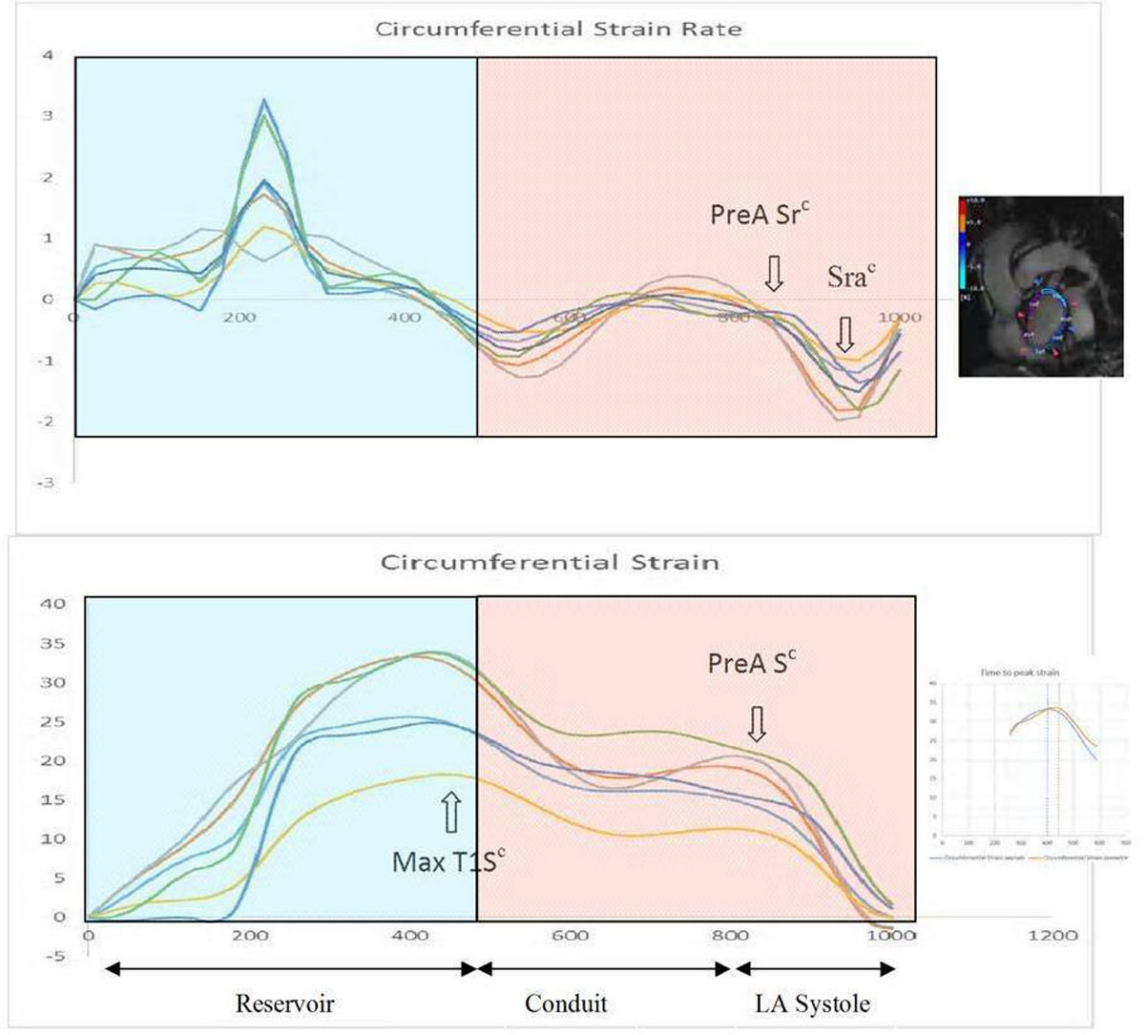
LA circumferential (panel A) and longitudinal (panel B) strain rate curves during a cardiac cycle using multimodality tissue tracking.

We performed a nested case-control analysis with 1:2 matching for 39 cases of paroxysmal AF (n=28, in sinus rhythm during cardiac magnetic resonance (CMR)) and HF (n=14, AF+HF; n=3) and 78 controls with similar demographic and clinical characteristics at the baseline (Table 1). LA circumferential (short axis) and longitudinal strain rate (horizontal long axis) were measured using Multi-modality Tissue Tracking (Toshiba, Japan) from short and long-axis cine CMR images. Circumferential LA systolic dyssynchrony among 18 LA segments (6 segments x 3 slices) was evaluated as; Standard Deviation (SD) of time to pre atrial contraction Strain rate (PreA Sr^c^) and Peak systolic strain rate (Peak Sra^c^) (Figure 1). Similarly, longitudinal LA dyssynchrony parameters (among 6 segments) were: SD-Time to pre-atrial contraction strain rate (PreA Sr^L^) and SD-Time to peak systolic strain rate (Peak-Sra^L^). Wilcoxon-rank sum test (non-parametric) or two sample t-test (parametric) were used for comparison between the groups.

**Table 1:** Left atrial circumferential and longitudinal systolic dyssynchrony parameters among the cases and the control group.

Parameters	Controls (n=78)			Cases (n=39) (paroxysmal AF + Heart Failure)			p-value
**Longitudinal**	**Mean ±SD**	**Median**	**IQR**	**Mean ±SD**	**Median**	**IQR**	

SD-TP PreA SrL, msec	36.43 ± 33.53	27.40	15.50 - 46.13	51.62 ± 33.40	39.70	32.07 - 61.78	0.001

SD-TP Peak SraL	35.92 ± 43.22	23.86	16.32 - 35.60	45.23 ± 42.04	32.50	22.98 - 44.54	0.027

**Circumferential**							

SD-TP PreA Src, msec	28.73±13.90	26.23	19.70 - 34.98	45.06 ± 30.25	35.49	24.84 - 57.03	0.010

SD-TP Peak Srac, msec	28.95 ± 23.1	23.04	16.46 - 31.57	36.46 ± 30.93	26.83	17.45 - 44.02	0.316

**LA maximum volume/BSA**	33.79 ± 9.57	31.72	28.64 - 38.86	45.02 ± 17.85	44.38	31.04 - 56.44	<0.0001

**Sra (%/ms)**	-1.85 ± 0.85	-1.74	-2.18: -1.45	-1.03 ± 0.61	-1.04	-1.04: -0.53	<0.0001

Participants were matched for: Age, gender, race/ethnicity.

## Results

In participants during MESA exam 5 (age 74±8 years, 51.4% men), systolic circumferential dyssynchrony (SD-TP-PreA Sr^c^, msec) was significantly higher in the cases compared to controls (45.06 vs. 28.73, p<0.010). Similarly, case group had greater longitudinal dyssynchrony than controls; SD-TP PreA Sr^L^ (51.62 vs. 36.43, p=0.001) and SD-TP-Peak Sra^L^ (45.23 vs. 35.92, p=0.027) (Table 1).

## Conclusions

Patients with paroxysmal atrial fibrillation and heart failure have significantly higher LA circumferential and longitudinal systolic dyssynchrony compared to normal controls.

## Funding

N/A.

